# Dyadic coping in patients with autoimmune diseases and spouses: a latent profile analysis and nursing implications

**DOI:** 10.3389/fpubh.2026.1883027

**Published:** 2026-08-10

**Authors:** Zheyuan Xia, Xiao Wang, Qiao Hu, Xiang Wang, Yukuan Miao

**Affiliations:** 1 The School of Nursing, Anhui University of Chinese Medicine, Hefei, China; 2 Laboratory of Geriatric Nursing and Health, Anhui University of Chinese Medicine, Hefei, China; 3 Emergency Intensive Care Unit (EICU), The First Affiliated Hospital of Anhui Medical University, Hefei, China

**Keywords:** autoimmune Diseases, couples, dyadic coping, influencing factors, latent profile analysis

## Abstract

**Objective:**

To identify the latent profiles of dyadic coping in patients with autoimmune diseases and their spouses, and to analyze the influencing factors associated with different profiles, thereby providing a theoretical basis for developing couple-based collaborative intervention programs.

**Methods:**

A cross-sectional survey design was used to conveniently recruit 262 dyads of patients with autoimmune diseases and their spouses. Demographic and disease-related data were collected using a self-designed general information questionnaire. Single three-level self-reported items (mild/moderate/severe) within this questionnaire were used to measure patients’disease-related economic burden and spouses’caregiving burden. Patients and their spouses completed the Dyadic Coping Inventory (DCI) and the Illness Perception Questionnaire. The Marital Adjustment Scale was additionally administered to identify distinct dyadic coping latent profiles. Mplus version 8.7 was employed to conduct latent profile analysis to identify different profiles of dyadic coping. SPSS version 27.0 was used for univariate analysis and multivariate logistic regression to explore factors associated with class membership.

**Results:**

A total of 282 questionnaires were distributed, and 262 valid dyads were included in the analysis, yielding an effective response rate of 92.91%. Four latent profiles of dyadic coping in patients and their spouses were identified via latent profile analysis: High Dyadic Coping Profile (Class 1, *n* = 39, 14.9%), Moderate Dyadic Coping Profile (Class 2, *n* = 118, 45%), Spouse-Moderate/Patient-Low Dyadic Coping Profile (Class 3, *n* = 58, 22.1%), and Patient-Moderate/Spouse-Low Dyadic Coping Profile (Class 4, *n* = 47, 18%). The entropy value of the model was 0.941, indicating good classification accuracy. Multinomial logistic regression analysis showed that multiple factors were significantly associated with membership in different dyadic coping subgroups. Using the High Dyadic Coping Profile as the reference category, illness perception was a strong and consistent positive predictor for all non-high-level subgroups (OR ranging from 1.683 to 3.188, all *p* < 0.001). Marital adjustment of both patients and spouses (OR ranging from 0.704 to 0.949, all *p* < 0.05) and mild economic/caregiving burden (OR ranging from 0.004 to 0.113, all *p* < 0.05) were protective factors for the different subgroups. Additionally, patient age was a positive predictor for the Patient-Moderate/Spouse-Low Dyadic Coping Profile (OR = 1.323, *p* < 0.05), whereas spouse age was a negative predictor for this profile (OR = 0.662, *p* < 0.05).

**Conclusion:**

Four heterogeneous subtypes of dyadic coping were identified among couples affected by autoimmune diseases, with 40.1% of couples exhibiting asymmetric imbalanced coping patterns. Illness perception emerged as the strongest predictor of imbalance, whereas marital adjustment served as a protective factor. The influence of economic and caregiving burden on dyadic coping exhibited a threshold effect: mild burden was protective, whereas moderate-to-severe burden may be associated with persistent coping impairment that is difficult to reverse. Clinical practice should implement stratified, targeted couple-based interventions according to the characteristics of each subtype.

## 1 Introduction

Autoimmune diseases are characterized by complex etiologies, protracted disease courses, and frequent involvement of multiple organ systems, resulting in persistent pain and functional limitations that necessitate long-term treatment (1). The World Health Organization classifies rheumatoid arthritis, systemic lupus erythematosus, Sjögren‘s syndrome, and systemic sclerosis as diffuse connective tissue diseases, all of which fall within the spectrum of autoimmune disorders (2). Among these, rheumatoid arthritis primarily presents as symmetrical joint erosion and persistent joint pain, progressively leading to joint deformity and functional disability; systemic lupus erythematosus is often marked by periodic acute flares affecting multiple organs including the kidneys, heart, and lungs, which significantly increase the risk of poor prognosis; Sjögren’s syndrome is characterized predominantly by chronic glandular damage, frequently accompanied by generalized dull pain and persistent fatigue; and systemic sclerosis is defined by progressive fibrosis, with gradual involvement of the skin, gastrointestinal tract, heart, and lungs (2). None of these conditions are curable, and patients require lifelong regular medication; the repeated flares, progressive functional decline, and long-term organ damage impose sustained chronic physical and psychological stress on patients. A retrospective cohort study with an 18-year follow-up demonstrated that this population faces a significantly higher risk of mental disorders than the general population (3): the standardized incidence ratios for systemic lupus erythematosus, rheumatoid arthritis, Sjögren‘s syndrome, and systemic sclerosis were 2.37, 1.84, 2.09, and 3.05, respectively. The physical and psychological distress caused by these diseases predisposes patients to prolonged negative emotions, which may further transmit to their spouses as primary caregivers, leading to anxiety, depression, and other psychological stress responses under long-term caregiving burden (4), and in turn, reciprocally affect patients’ disease adaptation and recovery.

The systemic transactional model of stress and coping (5) posits that patients and their spouses under disease-related stress are not isolated from each other, but rather constitute an interdependent, dynamically linked dyadic adaptive whole. Under this framework, dyadic coping refers to the processes by which couples jointly or reciprocally communicate about stress, provide support, and appraise and manage illness-related challenges (4, 5), representing the core couple-level construct of interest in this study. Autoimmune diseases, as chronic stressors, impose prolonged and heavy physical and psychological burdens on both partners. Systematic reviews and meta-analyses, using stroke as an example, have confirmed that effective dyadic coping can alleviate patients’ symptom burden, sustain marital intimacy, and enhance the overall quality of life for both partners (6). However, even under similar disease circumstances, significant heterogeneity exists across couples in the levels and combined patterns of dyadic coping. The literature suggests that this differentiation can be attributed to the continuous interaction of three factors throughout the disease trajectory. First, there are discrepancies in illness perception between patients and spouses: patients evaluate symptoms based on their own lived experience, whereas spouses tend to assess from a caregiver’s perspective, leading to frequent misalignment in the prioritization of coping needs (7). Second, differences in relationship resources such as marital adjustment, alongside varying levels of perceived family burden, jointly constrain each partner’s coping capacity (8). Third, dyadic coping itself is characterized by interactive and reciprocal dynamics, wherein one partner’s coping behaviors continually shape the other’s subsequent responses; the cumulative effects of these interactions become repeatedly reinforced over long-term disease management, ultimately resulting in heterogeneous coping patterns across couples (9).

A meta-analysis based on the Actor–Partner Interdependence Model (APIM) (10) confirmed that dyadic coping exhibits stable actor effects (*b* ≈ 0.33) and partner effects (*b* ≈ 0.18–0.20), indicating that the coping behaviors of both partners interact with each other and jointly influence marital satisfaction. The study also found that disease type and population characteristics moderate the strength of these associations, providing empirical evidence for the heterogeneity of dyadic interaction patterns among couples with chronic illnesses. Notably, all primary studies included in this meta-analysis relied on the total score of the Dyadic Coping Inventory to assess the overall level of dyadic coping; however, a single total score cannot capture the subtype stratification within the population. At the theoretical level, the “we-disease” concept proposed by Leuchtmann and Bodenmann (11) explicitly states that when one partner suffers from a severe physical or mental disorder, both partners are profoundly affected by the event, and the illness should be regarded as a shared experience of the couple rather than an isolated individual event. Nevertheless, most current studies have separated patients and spouses into independent samples for individual-level analyses (12), making it difficult to characterize the combined patterns and interactive dynamics of both partners’ coping behaviors. These methodological limitations hinder the effective interpretation of heterogeneity in couples’ disease-adaptation outcomes and constrain the development of stratified, targeted couple-based collaborative intervention programs.

Latent Profile Analysis (LPA) is grounded in a probabilistic model framework, which objectively determines the optimal number of latent classes through multiple goodness-of-fit indices and assigns individuals to classes based on posterior probabilities, thereby circumventing the subjective bias inherent in traditional arbitrary grouping methods (13). Its core advantage lies in treating the couple dyad as a complete analytical unit, simultaneously incorporating multi-dimensional coping indicators from both partners to precisely identify distinct coping subtypes. This method has been applied in various cancer populations: a latent class analysis of couples facing prostate cancer identified four subtypes: Efficient Coping, Ambivalent Coping, Stable Coping, and Coping Distress (14); studies on breast cancer couples also identified four subtypes: low-level coping group, medium-high common coping group, medium-high supportive coping group, and high-level coping group (15); and couples with gynecological cancers exhibited three coping profiles: low-DC, middle-DC, and high-DC groups (16). These findings indicate that heterogeneous coping subtypes exist among couples with cancer, and factors such as disease stage, couple communication quality, and self-efficacy significantly predict subtype membership.

However, empirical research on dyadic coping remains scarce for the four connective tissue diseases examined in this study, namely rheumatoid arthritis, systemic lupus erythematosus, Sjögren’s syndrome, and systemic sclerosis. Although analogous findings have been documented in a related but distinct group, namely neurological autoimmune conditions including neuromyelitis optica spectrum disorders (NMOSD) and myelin oligodendrocyte glycoprotein antibody-associated disease (MOGAD) (17), direct investigations targeting connective tissue disorders are still limited. These prevalent chronic conditions demand lifelong management, exposing patients and spouses to persistent stressors such as disease progression and recurrent flares. Unlike certain cancers that have a defined treatment endpoint, autoimmune diseases are characterized by lifelong disease burden and unpredictable relapses, making the stressors faced by couples more sustained and less predictable. Therefore, whether the subtype differentiation patterns and predictive factors that have been confirmed in the aforementioned cancer populations are applicable to couples affected by autoimmune diseases remains unclear. Accordingly, this study recruited patients diagnosed with the four aforementioned autoimmune diseases and their spousal caregivers. The primary objective was to conduct an exploratory latent profile analysis (LPA) to identify distinct dyadic coping profiles among couples. The secondary objective was to compare subgroup characteristics and identify predictors that distinguish these profiles. By achieving these objectives, this study aims to provide empirical evidence for developing targeted couple-based intervention strategies tailored to distinct coping profiles.

## 2 Subjects and methods

### 2.1 Study design and participants

This study employed a cross-sectional survey design. Between January 2025 and March 2026, participants were recruited from the outpatient clinics and inpatient wards of the rheumatology and immunology departments in three tertiary grade A hospitals in Anhui Province, China, using a convenience sampling method.

Inclusion criteria: (1) a confirmed diagnosis of one of the following four autoimmune diseases: systemic lupus erythematosus (SLE), systemic sclerosis (SSc), rheumatoid arthritis (RA), or Sjögren’s syndrome (SS). Patients with overlapping syndromes (e.g., coexisting RA and secondary SS) were excluded to ensure a clear classification of disease type. The specific diagnostic criteria were as follows: SLE according to the 2019 European League Against Rheumatism/American College of Rheumatology (EULAR/ACR) classification criteria (18); SSc according to the 2013 American College of Rheumatology/European League Against Rheumatism (ACR/EULAR) classification criteria (19); RA according to the 2010 ACR/EULAR classification criteria (20); and SS according to the 2016 ACR/EULAR classification criteria for primary Sjögren’s syndrome (21). (2) age ≥ 18 years; (3) disease duration ≥ 1 year (ensuring that patients had experienced sustained illness burden associated with their condition); (4) intact consciousness and basic abilities to read, comprehend, and communicate; and (5) provision of informed consent and voluntary participation in the study.

Exclusion criteria: (1) presence of other severe autoimmune diseases, malignant tumors, or serious cardiovascular or cerebrovascular diseases; or being in the acute phase of the disease (e.g., severe pulmonary infection, acute renal crisis); (2) severe dependence in activities of daily living, as assessed by the Barthel Index (22) (Barthel Index score ≤ 40); (3) experience of a traumatic event within the past year, such as severe natural disasters, accidents, or the death of a family member; (4) previous or current receipt of psychological intervention.

The sample size requirement for LPA primarily depends on class separation (effect size), the number of classes, and the number of indicators, rather than a simple rule of thumb based on the number of observed variables. Monte Carlo simulation studies by Tein et al. (23) demonstrated that when class separation is large (Cohen‘s d ≥ 0.8), a sample size of 200–300 is sufficient to achieve adequate power for class enumeration. Simulation studies by Nylund et al. (13) also indicated that the Bootstrap Likelihood Ratio Test (BLRT) performs well with a sample size of 200. Given the absence of prior parameters from similar studies, we reference the above methodological recommendations (sample size of 200–300 meets power requirements) and, taking into account the effective questionnaire recovery rate from our pilot study (≥90%), plan to enroll 270–300 patient-spouse dyads in couples affected by autoimmune diseases.

### 2.2 Study procedure

Our research team consisted of 7 members, taking on responsibilities in research management, quality control, data collection, and statistical analysis. All data collectors received unified standardized training, and simulated assessment exercises were conducted prior to formal data collection to ensure consistent survey implementation and enhance study reproducibility. All members had previous experience in community-based epidemiological surveys and received standardized training prior to the study, covering questionnaire interpretation, communication skills, and data entry protocols. Only those who passed a mock survey assessment were allowed to participate in the formal investigation.

All participants provided written informed consent. For those who were unable to read independently, the consent form was read aloud by the investigator, and a fingerprint was used in place of a signature in the presence of an independent witness. The survey was conducted in a quiet hospital ward or at the patient’s home, using a one-on-one questionnaire format, with completion time controlled between 15 and 25 min. Patients and their spouses were required to complete all study questionnaires, including the DCI, independently in separate quiet private spaces during on-site data collection. Neither partner was permitted to view the other’s answers at any stage. For participants with poor literacy or cognitive barriers, standardized neutral guidance was provided one-on-one by trained researchers without allowing mutual communication between the couple to avoid response contamination.

Within 24 h after questionnaire collection, data were reviewed and entered. Ambiguous information was promptly verified. Questionnaires with a response rate of less than 90%, completion time of less than 10 min, or showing patterned responses were excluded. Data were entered independently by two individuals to ensure accuracy. For minor residual missing values (<10%) retained in valid questionnaires, multiple imputation using chained equations (under the missing at random assumption) was applied prior to statistical analysis.

### 2.3 Variables and instruments

Potential influencing factors were screened via systematic literature review and team discussion. Two categories of variables were incorporated into the analytical model: core latent constructs and clinical predictive covariates. The core latent constructs comprised dyadic coping, illness perception, and marital adjustment. The clinical predictive covariates included sociodemographic and clinical characteristics collected by the self-designed general information questionnaire, namely age, education, marriage duration, disease subtype, disease duration, patient-reported economic burden, and spouse-reported caregiving burden.

The Barthel Index was administered by trained researchers during the initial screening phase to assess patients’ activities of daily living, with a score ≤ 40 serving as the exclusion threshold.

#### 2.3.1 Demographic and clinical instrument

A self-designed general information questionnaire was developed for both patients and their spouses on the basis of prior relevant literature. For patients, the questionnaire collected demographic and disease-related indicators: gender, age, educational attainment, employment status, disease type (rheumatoid arthritis, systemic lupus erythematosus, Sjögren’s syndrome, systemic sclerosis), disease duration (in years), and disease-related economic burden was assessed via a single self-reported item rated as mild, moderate, or severe, defined by the participant’s subjective perception of financial strain experienced over the preceding 6 months (mild = minimal financial impact; moderate = noticeable but manageable impact; severe = major impact with significant lifestyle changes). For spouses, collected variables included gender, age, education, employment, marriage duration, and caregiving burden, the latter also measured by a single independent item with the same three-level rating, defined by the spouse’s perceived intensity of caregiving demands over the same period.

Standardized written definitions were presented alongside each response option. For disease-related economic burden: mild indicated negligible extra household expenditure; moderate meant noticeable but manageable financial pressure; severe represented heavy medical costs disrupting the family’s regular living standard. For caregiving burden reported by spouses: mild referred to light, occasional tasks without emotional strain; moderate denoted regular daily care with mild psychological fatigue; severe described round-the-clock caregiving with persistent stress and impaired personal life.

#### 2.3.2 Dyadic coping inventory (DCI)

The Dyadic Coping Inventory (DCI) was developed by Bodenmann et al. (24) and translated and validated into Chinese by Xu et al. (25), yielding a full 37-item Chinese version. Subsequently, the research team conducted factor verification based on Chinese couple samples and derived a shortened 31-item five-factor version. To maintain the complete construct of stress communication, the shortened version was not adopted in the present study. All 37 original translated items were retained, and the six-dimensional theoretical framework proposed by Bodenmann et al. (24) was followed. The scale consists of six dimensions: stress communication (8 items), supportive dyadic coping (10 items), delegated dyadic coping (4 items), negative dyadic coping (8 items, reverse-scored), joint dyadic coping (5 items), and evaluation of dyadic coping (2 items). The dimensions of stress communication, supportive dyadic coping, delegated dyadic coping, and negative dyadic coping cover both self-perceived and partner-perceived coping, while the joint dyadic coping dimension reflects couples’ perceived collaborative coping behaviors when both partners experience stress simultaneously. The two items of evaluation of dyadic coping serve only as reference indicators of satisfaction and are excluded from the total score. Items are rated on a 5-point Likert scale ranging from 1 (“very rarely”) to 5 (“very frequently”). Negative coping items are reverse-scored. The two items assessing coping quality are not included in the total score. For each dimension used as indicators in latent profile analysis (LPA), all self-perceived and partner-perceived items within the same dimension were combined to calculate dimension average scores, which were computed as the total score of the dimension divided by the number of items in that dimension. These dimension average scores were then input into the LPA as corresponding indicators (H1–H5 and P1–P5). The total score ranges from 35 to 175, with scores <111 indicating low-level couple coping, 111–145 indicating moderate-level, and >145 indicating high-level; higher scores represent higher levels of dyadic coping. In this study, the Cronbach’s *α* coefficients for the subscales ranged from 0.821 to 0.916.

#### 2.3.3 Brief illness perception questionnaire (BIPQ)

The BIPQ was developed by Broadbent et al. (26) to rapidly assess patients’ subjective cognitive and emotional representations of their illness. The scale comprises 8 items across three dimensions: cognitive representations (5 items), emotional representations (2 items), and illness comprehensibility (1 item). Items are rated on an 11-point Likert scale ranging from 0 (“not at all”/“no effect at all”) to 10 (“extremely”/“very severely”), yielding a total score between 0 and 80. Higher scores indicate more severe perceived illness symptoms and more negative illness perceptions. In this study, the Cronbach’s *α* coefficient for the scale was 0.79.

#### 2.3.4 Locke–Wallace marriage adjustment test (LWMAT)

The LWMAT was developed by American psychologist Locke et al. (27). The scale consists of 15 items that assess marital intimacy across four dimensions: marital satisfaction, emotional expression, couple cohesion, and couple consensus. The scale was completed independently by patients and their spouses. In this study, the Cronbach’s *α* coefficient was 0.82 for the patient version and 0.81 for the spouse version.

### 2.4 Statistical analysis

Data entry was performed using Epidata version 3.1, and data analysis was conducted using SPSS version 27.0. Continuous variables were expressed as mean ± standard deviation, and categorical variables as frequencies and percentages. One-way ANOVA and chi-square tests were used to examine differences in demographic and clinical variables across latent profiles. For variables with significant overall group differences, Bonferroni-corrected pairwise post-hoc multiple comparisons were further performed to identify specific inter-profile differences. The corresponding pairwise comparison results were marked with superscripts in Table 1 according to predefined group contrast rules, with a family-wise error rate controlled at *α* = 0.05 using Bonferroni correction.

**Table 1:** General information and dyadic coping scores of patients with autoimmune diseases and their spouses (*n* = 262).

Variables	Overall (*n* = 262)	High dyadic coping profile (*n* = 39)	Moderate dyadic coping profile (*n* = 118)	Spouse-moderate/Patient-low dyadic coping profile(*n* = 58)	Patient-moderate/Spouse-low dyadic coping profile(*n* = 47)	*F*/*X*^2^	*p* value
Patient							
Gender						0.266^1)^	0.966
Female	223(85.1)	33(84.6)	99(83.9)	50(84.7)	41(87.5)		
Male	39(14.9)	6(15.4)	19(16.1)	8(15.3)	6(12.5)		
Age(year)	48.86 ± 9.19	51.08 ± 8.72	50.71 ± 9.22	49.02 ± 7.98	42.42 ± 8.12ᶜᵉᶠ	11.417^2)^	<0.001
Education						4.345^1)^	0.227
High school or below	147(56.1)	19(48.7)	72(61)	35(61.0)	21(44.7)		
College or above	115(43.9)	20(51.3)	46(39)	23(39.0)	26(55.3)		
Employment status						3.772^1)^	0.287
Employed	188(71.8)	26(66.7)	82(69.5)	46(79.3)	34(72.3)		
Unemployed/retired	74(28.2)	13(33.3)	36(30.5)	12(20.7)	13(27.7)		
Disease type						7.941^1)^	0.540
RA	98(37.6)	16(41.0)	45(38.1)	22(37.9)	15(31.9)		
SLE	42(16.0)	7(17.9)	16(13.6)	8(13.8)	11(23.4)		
SSC	27(10.3)	6(15.4)	8(6.8)	8(13.8)	5(10.6)		
Sjögren’s syndrome	95(36.3)	10(25.6)	49(41.5)	20(34.5)	16(34.0)		
Disease duration						4.529^1)^	0.606
1–4 year	210(80.2)	30(76.9)	95(80.5)	46(79.3)	39(83.0)		
5–10 year	31(11.8)	7(17.9)	11(9.3)	9(15.5)	4(8.5)		
≥11 year	21(8.0)	2(5.2)	12(10.2)	3(5.2)	4(8.5)		
Economic burden						51.555^1)^	<0.001
Mild	69(26.3)	24(61.5)^bc^	34(28.8)^de^	5(8.6)^bdf^	6(12.8)^ce^ᶠ		
Moderate	121(46.2)	9(23.1)^bc^	64(54.2)^de^	24(41.4)^bd^	24(51.1)^ce^		
Severe	72(27.5)	6(15.4)^bc^	20(17.0)^de^	29(50.0)^bdf^	17(36.1)^cef^		
Dyadic coping score	121.40 ± 14.57	148.38 ± 6.72^abc^	121.53 ± 6.09^ae^	104.61 ± 6.54^bdf^	119.81 ± 6.21^cf^	378.21^2)^	<0.001
Stress communication dimension	12.43 ± 2.79	16.79 ± 1.56^abc^	12.63 ± 1.55^ade^	11.92 ± 2.26bdf	9.54 ± 1.76c^ef^	137.061	<0.001
Supportive coping dimension	32.10 ± 4.41	38.26 ± 2.71^abc^	32.42 ± 3.58^ae^	31.75 ± 2.96^bf^	27.66 ± 2.11^cef^	94.405	<0.001
Delegated coping dimension	16.29 ± 3.61	22.82 ± 2.39^abc^	16.22 ± 1.60^ade^	15.35 ± 2.48^bd^	12.90 ± 1.96^ce^	199.936	<0.001
Negative coping dimension	31.73 ± 3.56	36.46 ± 2.16^abc^	32.11 ± 1.97^e^	31.31 ± 3.10^bf^	28.19 ± 3.20^cef^	84.392	<0.001
Joint coping dimension	28.86 ± 3.05	34.05 ± 1.75^abc^	28.16 ± 2.22^ae^	29.48 ± 1.37^df^	26.32 ± 1.59cef	141.192	<0.001
Illness perception score	46.32 ± 4.38	41.15 ± 2.36^abc^	45.37 ± 3.02^ade^	47.76 ± 2.54^bdf^	51.04 ± 4.69^cef^	75.353^2)^	<0.001
Marital Adjustment score	124.37 ± 19.51	143.54 ± 12.05^abc^	128.34 ± 18.84^ade^	117.25 ± 16.24^bdf^	107.96 ± 10.97^cef^	40.99^2)^	<0.001
Spouse							
Gender						0.266^1)^	0.966
Male	223(85.1)	33(84.6)	99(83.9)	50(84.7)	41(87.5)		
Female	39(14.9)	6(15.4)	19(16.1)	8(15.3)	6(12.5)		
Age(year)	47.98 ± 9.77	53.90 ± 9.29	50.00 ± 8.28	49.49 ± 6.70	36.42 ± 7.61^cef^	43.75^2)^	0.001
Education						2.114^1)^	0.549
High school or below	118(45.0)	21(53.8)	52(44.1)	27(46.6)	18(38.3)		
College or above	144(55.0)	18(46.2)	66(55.9)	31(52.5)	29(61.7)		
Employment status						6.810^1)^	0.078
Employed	187(71.4)	24(61.5)	82(69.5)	41(70.7)	40(85.1)		
Unemployed/retired	75(28.6)	15(38.5)	36(30.5)	17(29.3)	7(14.9)		
Marriage duration	23.60 ± 9.39	28.92 ± 8.89^abc^	26.46 ± 8.61^ade^	22.34 ± 6.89^bd^	13.90 ± 6.60^cef^	35.119^2)^	<0.001
Caregiving burden						33.393^1)^	<0.001
Mild	80(30.5)	22(56.4)^bc^	36(30.5)^e^	16(27.7)^b^	6(12.8)^cef^		
Moderate	88(33.6)	11(28.2)^bc^	42(35.6)	25(43.1)^b^	10(21.3)^ce^		
Severe	94(35.9)	6(15.4)^bc^	40(33.9)^e^	17(29.3)^bf^	31(65.9)^cef^		
Dyadic coping score	124.21 ± 16.26	151.21 ± 6.64^abc^	126.06 ± 5.45^ac^	123.08 ± 5.99^bf^	99.19 ± 7.88^cef^	502.78^2)^	<0.001
Stress communication dimension	13.88 ± 2.72	18.26 ± 1.82^abc^	14.22 ± 1.81^ade^	12.25 ± 1.65^bdf^	11.50 ± 1.37^cef^	136.895	<0.001
Supportive coping dimension	34.73 ± 4.08	39.38 ± 3.09^abc^	35.81 ± 2.33^ade^	34.51 ± 2.26^bdf^	28.63 ± 2.51^cef^	151.176	<0.001
Delegated coping dimension	18.48 ± 3.55	23.51 ± 1.70^ac^	18.22 ± 2.10^ad^	19.51 ± 1.63^bd^	13.75 ± 2.89^cef^	157.546	<0.001
Negative coping dimension	28.77 ± 5.25	35.59 ± 2.40^abc^	29.76 ± 2.49^e^	29.46 ± 2.59^bf^	20.00 ± 2.24^cef^	311.841	<0.001
Joint coping dimension	28.35 ± 3.71	34.46 ± 2.11^abc^	28.05 ± 1.61^ac^	27.36 ± 2.18^bf^	25.31 ± 4.31^cef^	104.792	<0.001
Marital Adjustment score	120.76 ± 18.65	140.74 ± 9.32ᵃᵇᶜ	128.10 ± 11.98ᵃᵈᵉ	113.37 ± 11.33ᵇᵈᶠ	95.83 ± 13.58ᶜᵉᶠ	131.428	P < 0.001

1)*X*^2^.

2) *F*.

Superscripts a–f represent significant pairwise contrasts after Bonferroni correction (*α* = 0.05): a = High vs Moderate, b = High vs Spouse-Moderate/Patient-Low, c = High vs Patient-Moderate/Spouse-Low, d = Moderate vs Spouse-Moderate/Patient-Low, e = Moderate vs Patient-Moderate/Spouse-Low, f = Spouse-Moderate/Patient-Low vs Patient-Moderate/Spouse-Low. Absence of superscript indicates non-significance between the corresponding pair. Detailed descriptions of pairwise contrasts are provided in the Results section.

Latent profile analysis was performed using Mplus 8.7. Given the inherent interdependence between patient and spouse data, we conducted dyadic latent profile analysis (DLPA) with the couple dyad as the unit of analysis. Scores from the five dimensions of the Dyadic Coping Inventory for both patients and their spouses were entered simultaneously as manifest indicators. To explicitly account for dyadic interdependence, we allowed within-profile covariances between corresponding patient and spouse indicators (i.e., H1 with P1, H2 with P2, … H5 with P5), thereby relaxing the conditional independence assumption for these paired indicators. We sequentially estimated models containing one to five latent profiles. Model fit was evaluated using the Akaike Information Criterion (AIC), Bayesian Information Criterion (BIC), sample-size-adjusted BIC (aBIC), entropy, the Lo–Mendell–Rubin adjusted likelihood ratio test (LMRT), and the bootstrapped likelihood ratio test (BLRT) (13). Lower AIC, BIC, and aBIC values indicated better model fit. Entropy reflects classification accuracy, ranging from 0 to 1, with values ≥ 0.80 indicating classification accuracy over 90% (23). The LMRT and BLRT compared the n-profile model against the *n* − 1-profile model; *p* < 0.05 suggested the n-profile model achieved significantly superior fit (28). The optimal number of profiles was determined by comprehensive evaluation of all fit indices combined with interpretability of each subgroup.

For multinomial logistic regression analysis, latent profile membership was set as the dependent variable. A unified theory-guided and statistically inclusive variable selection strategy was applied for model construction. Specifically, three core theoretical constructs derived from the systemic transactional model of stress and coping (illness perception, marital adjustment, and perceived family burden) were mandatorily included regardless of univariate statistical outcomes to preserve theoretical integrity. Meanwhile, additional demographic and spousal contextual variables were screened using a liberal univariate threshold of *p* < 0.20 combined with clinical relevance to avoid omitting potential confounders. The full set of candidate predictors covered patient demographic, clinical, psychological variables and spouse-related demographic, relational and burden indicators, including spouse employment status. Prior to model estimation, multicollinearity among continuous predictors was assessed via tolerance and variance inflation factor (VIF); VIF > 5 denoted severe collinearity. Marriage duration was excluded from the final model owing to severe collinearity with spouse age (VIF > 5). After this exclusion, no severe collinearity remained among the retained variables (all VIF < 5). The goodness-of-fit of conventional multinomial logistic regression was assessed using three pseudo-R^2^ statistics: Cox-Snell, Nagelkerke, and McFadden. Sparse categorical subgroups led to quasi-complete separation, extreme regression coefficients, distorted odds ratios and overly wide confidence intervals. To address this limitation, we reported cell frequencies for all categorical predictors alongside regression outputs. Moreover, Firth-type penalized multinomial logistic regression was implemented as sensitivity analysis to mitigate estimation bias under sparse-data conditions. We systematically compared results from standard and bias-corrected penalized models in terms of coefficient direction, statistical significance, and adjusted odds ratios with 95% CIs, and synthesized both sets of outputs for robust interpretation. A systematic comparison between standard and Firth-type penalized multinomial logistic regression was conducted as a sensitivity analysis. All core theoretical predictors (illness perception, patient marital adjustment, and spouse marital adjustment) exhibited consistent coefficient directions and identical statistical significance patterns, alongside highly similar odds ratio estimates in both model specifications. For categorical predictors with sparse cell frequencies (e.g., graded economic burden and spousal caregiving burden), Firth penalization produced the anticipated shrinkage of effect sizes toward 1, yet no predictor displayed reversed effect direction or changed substantive statistical interpretation. Owing to the high overall consistency of findings between the two estimation approaches, standard maximum-likelihood regression outputs were prioritized for the primary result table to prevent redundant content, while complete results from the bias-corrected Firth model were separately compiled for comparative inspection. All statistical tests adopted a two-sided significance level of *p* < 0.05. All categorical predictors (including patients’ economic burden, spousal caregiving burden, and spousal employment status) were coded with the highest-burden group and the unemployed-retired group set as reference categories, and detailed coding rules are shown in the corresponding supplementary table.

### 2.5 Ethical considerations

This study adhered to the ethical principles established in the Declaration of Helsinki. The research protocol was approved by the Ethics Committee of Anhui University of Chinese Medicine (Approval No.: AHUCM-HSS-2025008). All participants provided written informed consent after being fully informed of the study content and procedures. We adopted coded questionnaire data collection; fingerprints were collected only as biometric identifiers for participant verification. All collected data were used solely for statistical analysis, with participants’ privacy rights strictly protected throughout the whole research process.

## 3 Results

### 3.1 General information and dyadic coping scores of patients with autoimmune diseases and their spouses

A total of 282 questionnaires were distributed, and 262 valid dyads were returned, yielding an effective response rate of 92.91%. Among the participants, patient age ranged from 27 to 71 years, with a mean of 48.86 ± 9.19 years. There were 223 female patients (85.1%) and 39 male patients (14.9%). Regarding education level, the majority had high school education or below (147 patients, 56.1%). A total of 188 patients (71.8%) were employed. Two hundred and ten patients (80.2%) had a disease duration of 1–4 years. Seventy-two patients (27.5%) perceived their disease-related economic burden as severe. The mean illness perception score was 46.32 ± 4.38, and the mean marital adjustment score as perceived by patients was 124.37 ± 19.51.

Spouse age ranged from 25 to 73 years, with a mean of 47.98 ± 9.77 years. The mean marriage duration was 23.60 ± 9.39 years. The mean marital adjustment score as perceived by spouses was 120.76 ± 18.65. Ninety-four spouses (35.9%) perceived their caregiving burden as severe.

The total dyadic coping scores of patients with autoimmune diseases and their spouses were (121.40 ± 14.57) and (124.21 ± 16.26), respectively. Scores for each dimension of dyadic coping in patients and spouses, as well as the distribution of total scores across latent profiles, are presented in Table 1.

### 3.2 Model selection and determination of the optimal number of profiles

A person-centered latent profile analysis was conducted on the dyadic coping scores of 262 dyads of patients with autoimmune diseases and their spouses. One to five latent profile models were constructed based on dyadic coping assessment indicators, and detailed fit indices for all models are presented in Table 2.

**Table 2:** Fit indices of latent profile models for dyadic coping in patients with autoimmune diseases and their spouses (*n* = 262).

Model	AIC	BIC	aBIC	Entropy	*P* value		Class probability (%)
					LMRT	BLRT	
1-Profile	3976.799	4066.008	3986.747	—	—	—	100.000
2-Profile	3122.226	3250.686	3136.55	0.997	0.0785	*P* < 0.001	84.8/15.2
3-Profile	2566.744	2734.456	2585.445	0.993	0.0198	*P* < 0.001	66.7/18.1/15.2
4-Profile	2362.053	2569.017	2385.131	0.941	0.0047	*P* < 0.001	14.9/18 / 45/22.1
5-Profile	2240.356	2486.571	2267.81	0.948	*P* < 0.001	*P* < 0.001	0.3/17.9/21.9/44.9/14.9

aBIC, sample size-adjusted Bayesian information criterion; LMRT, Lo–Mendell–Rubin adjusted likelihood ratio test.

AIC, Akaike information criterion; BIC, Bayesian information criterion; BLRT, bootstrap likelihood ratio test.

*p* < 0.05: The *n*-category model is significantly superior to the *n*-l-category model.

The AIC, BIC, and aBIC information criteria decreased sequentially as the number of latent profiles increased, indicating continuous improvement in model fit. To precisely quantify model improvement between consecutive models, differences in core fit indices were compared. Compared with the 1-profile model, the 2-profile model showed substantial reductions in fit indices, with AIC decreasing by 854.573, BIC by 815.322, and aBIC by 850.197. The 3-profile model further reduced values relative to the 2-profile model, with decreases of 555.482 in AIC, 516.230 in BIC, and 551.105 in aBIC. The 4-profile model still achieved favorable improvement over the 3-profile model, with reductions of 204.691 in AIC, 165.439 in BIC, and 200.314 in aBIC. However, marginal improvement diminished substantially from the 4-profile to the 5-profile model, with only a 121.697 decrease in AIC, 82.446 in BIC, and 117.321 in aBIC, accompanied by a marked increase in model complexity.

All models from the 2-profile to 5-profile solution exhibited excellent classification accuracy, with all entropy values exceeding 0.80. Specifically, entropy values were 0.997 for the 2-profile model, 0.993 for the 3-profile model, 0.941 for the 4-profile model, and 0.948 for the 5-profile model, all reflecting high classification precision of latent grouping.

BLRT *p*-values for all 2 to 5-profile models were < 0.001, meaning each model yielded significantly better fit than its preceding nested model. The LMRT *p*-value for the 2-profile model was 0.0785 (*p* > 0.05), indicating no statistically meaningful improvement in fit relative to the 1-profile model. LMRT *p*-value for the 3-profile model was 0.0198, and the 4-profile model returned an LMRT *p*-value of 0.0047; the 5-profile model also returned a significant LMRT result (*p* < 0.001), demonstrating that the 3-, 4-, and 5-profile models fit significantly better than their respective nested models.

In terms of latent profile distribution, the 4-profile model generated balanced, clinically interpretable class probabilities of 14.9, 45.0, 22.1, and 18%. No subgroup accounted for less than 5% of the total sample, which avoided severely unbalanced classification. By contrast, the 5-profile model contained an extremely small subgroup representing merely 0.3% of participants, resulting in uneven grouping, unreliable profile characteristics, and limited clinical interpretability.

Taken together, the 1-profile model was overly simplistic. Although the 2-profile model had high classification accuracy, its LMRT result was non-significant (*p* = 0.0785), suggesting negligible improvement over the single-profile model. While the 5-profile model produced the lowest absolute information criterion values, it suffered from severe class imbalance and excessive complexity. Integrating the magnitude of fit index reductions, classification accuracy, significance of likelihood ratio tests, and substantive clinical interpretability, the 4-profile model was ultimately identified as the optimal latent profile solution for dyadic coping within patient–spouse dyads.

### 3.3 Characterization and labeling of the identified profiles

**Figure 1:**
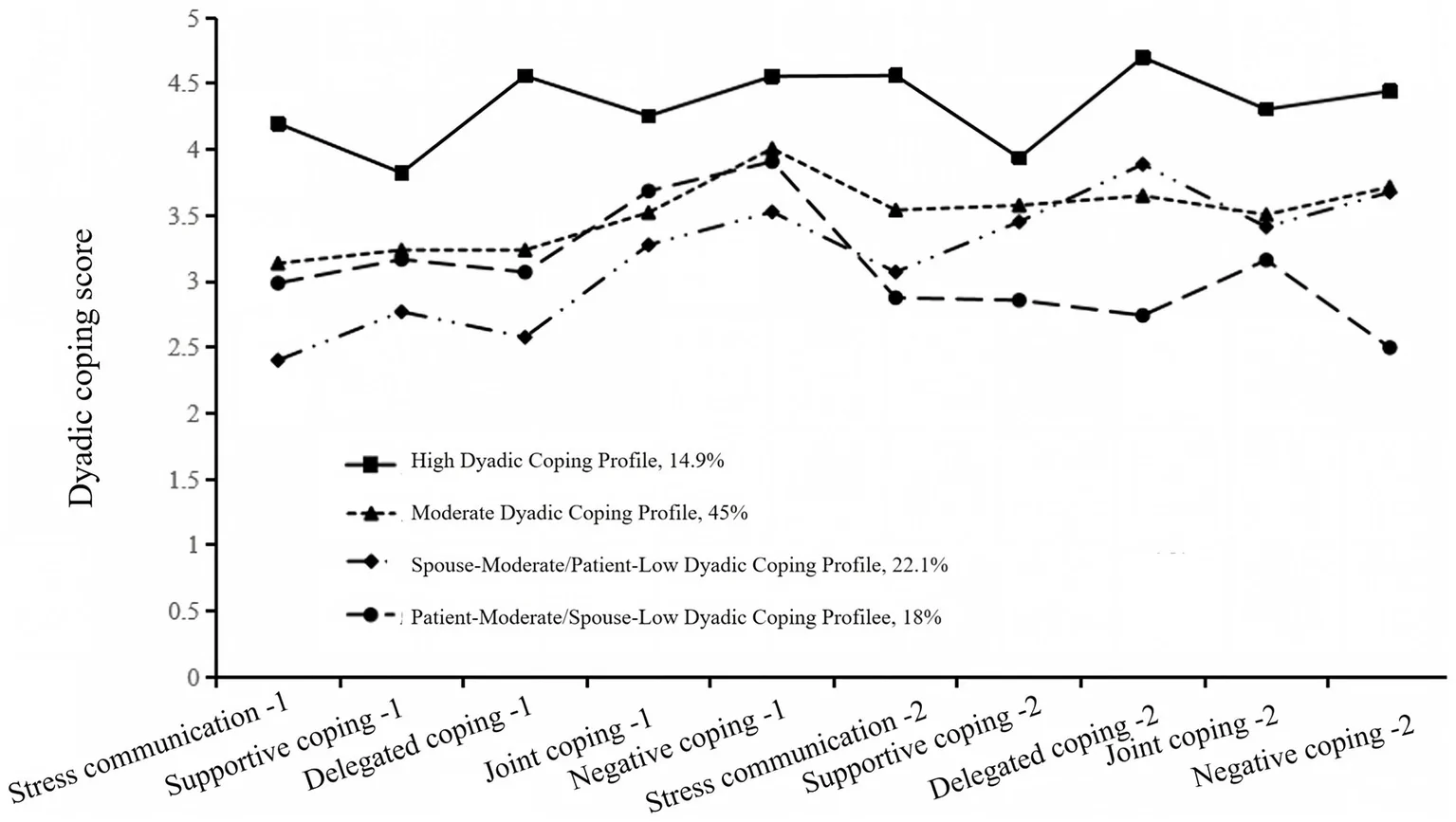
Latent profile plot of dyadic coping in couples with autoimmune diseases.

A latent profile plot of dyadic coping in patients with autoimmune diseases and their spouses (Figure 1) was generated to characterize the four latent profiles of dyadic coping. The four profiles were named according to their respective characteristics.

Profile 1 comprised 39 dyads (14.9%). The total dyadic coping score was 148.38 ± 6.72 for patients and 151.21 ± 6.64 for spouses. Both patients and spouses had relatively high mean item scores across all dimensions of dyadic coping, with total scores >145 for both, indicating high-level coping. This profile was therefore named the High Dyadic Coping Profile.

Profile 2 comprised 118 dyads (45%). The total dyadic coping score was 121.53 ± 6.09 for patients and 126.06 ± 5.45 for spouses. Both patients and spouses had moderate mean item scores across all dimensions, with total scores between 111 and 145 for both, indicating moderate-level coping. This profile was therefore named the Moderate Dyadic Coping Profile.

Profile 3 comprised 58 dyads (22.1%). The total dyadic coping score was 104.61 ± 6.54 for patients and 123.08 ± 5.99 for spouses. Patients had a total score <111 (low level), while spouses had a total score between 111 and 145 (moderate level). This profile was therefore named the Spouse-Moderate/Patient-Low Dyadic Coping Profile.

Profile 4 comprised 47 dyads (18%). The total dyadic coping score was 119.81 ± 6.21 for patients and 99.19 ± 7.88 for spouses. Patients had a total score between 111 and 145 (moderate level), while spouses had a total score <111 (low level). This profile was therefore named the Patient-Moderate/Spouse-Low Dyadic Coping Profile.

### 3.4 Results of univariate analysis of latent profiles of dyadic coping in patients with autoimmune diseases and their spouses

The results of univariate analysis showed that the four groups differed significantly in terms of patient age, economic burden, dyadic coping score, illness perception, marital adjustment, as well as spouse’s marriage duration, caregiving burden, spouse’s dyadic coping score, and spouse’s marital adjustment (all *p* < 0.05) (see Table 1).

Bonferroni-corrected pairwise comparisons further clarified the specific inter-group differences marked by superscripts a–f in Table 1.

High Dyadic Coping Profile exhibited significantly better indicators (higher dyadic coping and marital adjustment scores, milder economic and caregiving burden, older patient/spouse age, longer marriage duration, lower illness perception) than the other three profiles (marked with superscript a, b, c).

Compared with the Moderate Dyadic Coping Profile, the Spouse-Moderate/Patient-Low Dyadic Coping Profile had lower marital adjustment scores, heavier economic burden and poorer spousal coping status (superscript d).

The Patient-Moderate/Spouse-Low Dyadic Coping Profile showed the worst spousal coping and marital adjustment, younger patient and spouse age, heavier care burden, and higher patient illness perception relative to the Moderate group (superscript e).

The Patient-Moderate/Spouse-Low group had significantly lower spousal coping scores and heavier spousal care burden than the Spouse-Moderate/Patient-Low group (superscript f).

All Bonferroni-corrected pairwise comparisons corresponding to superscripts a to f are interpreted above, with significant differences visually denoted in Table 1.

### 3.5 Multinomial logistic regression analysis of factors influencing latent profiles of dyadic coping in patients with autoimmune diseases and their spouses

A multinomial logistic regression analysis was conducted to examine predictors of the four latent dyadic coping profiles. Initial independent variables included patient age, illness perception, patient marital adjustment, patient economic burden (severe burden as reference), spouse age, marriage duration, spouse marital adjustment, spouse caregiving burden (severe burden as reference), and spouse employment status (unemployed/retired as reference). The four latent dyadic coping profiles served as the dependent variable, with the High Dyadic Coping Profile designated as the reference group. Detailed variable coding and assignments are displayed in Table 3.

**Table 3:** Variable assignment.

Variable	Variable assignment
Patient age	Actual data
Illness perception	Actual data
Patient marital adjustment	Actual data
Patient economic burden	1 = Mild, 2 = Moderate, 3 = Severe (reference)
Spouse age	Actual data
Marriage duration	Actual data
Spouse marital adjustment	Actual data
Spouse employment status	1 = Employed, 2 = Unemployed/retired (reference)
Spouse caregiving burden	1 = Mild, 2 = Moderate, 3 = Severe (reference)

Before reporting independent predictors distinguishing latent profiles, the overall performance of the multinomial logistic regression model was assessed via pseudo-*R*^2^ statistics. The model demonstrated good overall fit, with Cox-Snell *R*^2^ = 0.801, Nagelkerke *R*^2^ = 0.867, and McFadden *R*^2^ = 0.626. Multicollinearity diagnostics were performed prior to model estimation, as detailed in Section 2.4. Marriage duration was excluded from the final model owing to severe collinearity with spouse age (VIF > 5). Spouse employment status was retained in the multivariate model without multicollinearity concern. All remaining predictors had tolerance values ranging from 0.250 to 0.917 and VIF values from 1.090 to 4.000, suggesting no severe multicollinearity. Using the high-level dyadic coping profile as the reference group, the multivariate analysis of latent dyadic coping profiles among couples with autoimmune diseases is displayed in Table 4.

**Table 4:** Multivariate analysis of latent profiles of dyadic coping in patients with autoimmune diseases and their spouses (*n* = 262).

VVariable	Moderate dyadic coping profile						Spouse-moderate/Patient-low dyadic coping profile						Patient-moderate/Spouse-low dyadic coping profile					
	*β*	Cell *n*	Odds ratio	95% CI		*p* value	*β*	Cell n	Odds ratio	95% CI		*p* value	*β*	Cell n	Odds ratio	95% CI		*p* value
				Lower	Upper					Lower	Upper					Lower	Upper	
Patient																		
Age	0.139	—	1.149	0.970	1.361	0.108	0.182	—	1.200	0.984	1.462	0.071	0.280	—	1.323	1.020	1.717	<0.05
Illness perception	0.521	—	1.683	1.298	2.182	<0.001	0.827	—	2.287	1.659	3.153	<0.001	1.159	—	3.188	1.884	5.393	<0.001
Marital adjustmen	−0.053	—	0.949	0.907	0.992	<0.05	−0.089	—	0.915	0.868	0.965	<0.001	−0.162	—	0.850	0.781	0.927	<0.001
Economic burden																		
Mild	−1.501	32/24	0.223	0.041	1.217	0.083	−4.192	6/24	0.015	0.002	0.151	<0.001	−4.714	7/24	0.009	0.001	0.238	<0.05
Moderate	0.292	64/9	1.339	0.249	7.205	0.734	−1.267	24/9	0.282	0.039	2.039	0.210	−1.825	24/9	0.161	0.010	2.729	0.206
Severe (ref)		20/6	1					29/6	1					17/6	1			
Spouse																		
Age	−0.162	—	0.850	0.707	1.023	0.085	0.179	—	1.196	0.934	1.532	0.155	−0.413	—	0.662	0.452	0.969	<0.05
Marital adjustment	−0.095	—	0.909	0.852	0.970	0.004	−0.182	—	0.834	0.771	0.902	<0.001	−0.350	—	0.704	0.621	0.799	<0.001
Caregiving burden																		
Mild	−1.526	34/22	0.218	0.042	1.137	0.071	−2.184	17/22	0.113	0.014	0.902	<0.05	−5.568	7/22	0.004	0.001	0.133	<0.05
Moderate	−0.762	42/11	0.467	0.078	2.786	0.403	−0.698	25/11	0.498	0.060	4.110	0.517	−2.346	10/11	0.096	0.006	1.665	0.107
Severe (ref)		40/6	1					17/6	1					31/6	1			
Spouse employment status																		
Employed	−0.296	80/24	0.744	0.472	1.138	0.201	−0.405	42/24	0.667	0.409	1.046	0.104	−0.529	41/24	0.589	0.342	0.972	0.063
Unemployed/retired (ref)		36/15	1					17/15	1					7/15	1			

Reference group = high dyadic coping profile.

Cell *n* denotes the sample size of each categorical subgroup vs. reference group.

For the Moderate Dyadic Coping Profile (compared with the High Dyadic Coping Profile): Lower patient marital adjustment and lower spouse marital adjustment were significant negative predictors (OR = 0.949, 95% CI: 0.907–0.992, *p* < 0.05; OR = 0.909, 95% CI: 0.852–0.970, *p* = 0.004, respectively), indicating that decreased marital adjustment in either partner increased the likelihood of belonging to the moderate coping group. Conversely, patient illness perception was a significant positive predictor (OR = 1.683, 95% CI: 1.298–2.182, *p* < 0.001), suggesting that stronger illness-related distress was associated with a higher probability of moderate (rather than high) dyadic coping. Patient age, economic burden, spouse age, and spouse caregiving burden were not statistically significant (all *p* > 0.05). Spouse employment status showed no significant association with profile membership (*p* = 0.201).

For the Spouse-Moderate/Patient-Low Dyadic Coping Profile (compared with the High Dyadic Coping Profile): Significant positive predictors included patient illness perception (OR = 2.287, 95% CI: 1.659–3.153, *p* < 0.001). Significant negative predictors included patient marital adjustment (OR = 0.915, 95% CI: 0.868–0.965, *p* < 0.001), mild patient economic burden (OR = 0.015, 95% CI: 0.002–0.151, *p* < 0.001), spouse marital adjustment (OR = 0.834, 95% CI: 0.771–0.902, *p* < 0.001), and mild spouse caregiving burden (OR = 0.113, 95% CI: 0.014–0.902, *p* < 0.05). Lower patient marital adjustment and lower spouse marital adjustment increased the risk of belonging to this profile. Compared with severe burden, mild economic burden and mild caregiving burden were protective factors, reducing the probability of belonging to this low-level imbalanced coping profile. Higher patient illness perception was associated with an increased risk of belonging to this profile. Spouse employment status was not a statistically significant predictor (*p* = 0.104).

For the Patient-Moderate/Spouse-Low Dyadic Coping Profile (compared with the High Dyadic Coping Profile): Significant positive predictors included patient age (OR = 1.323, 95% CI: 1.020–1.717, *p* < 0.05) and patient illness perception (OR = 3.188, 95% CI: 1.884–5.393, *p* < 0.001). Significant negative predictors included patient marital adjustment (OR = 0.850, 95% CI: 0.781–0.927, p < 0.001), mild patient economic burden (OR = 0.009, 95% CI: 0.001–0.238, *p* < 0.05), spouse age (OR = 0.662, 95% CI: 0.452–0.969, *p* < 0.05), spouse marital adjustment (OR = 0.704, 95% CI: 0.621–0.799, *p* < 0.001), and mild spouse caregiving burden (OR = 0.004, 95% CI: 0.001–0.133, *p* < 0.05). Lower patient marital adjustment, younger spouse age, and lower spouse marital adjustment increased the risk of belonging to this profile. Lighter patient economic burden and lighter spouse caregiving burden reduced the risk of belonging to this profile. Older patient age and higher patient illness perception were associated with a significantly increased risk of belonging to this profile. Spouse employment status exhibited a marginally non-significant association with profile allocation (*p* = 0.063).

To mitigate estimation bias caused by sparse categorical cells, Firth-type penalized multinomial logistic regression was further performed as a sensitivity analysis, with all complete penalized regression outputs presented in Table 5. Comparisons between Table 4 and Table 5 confirmed consistent predictor directions and identical statistical significance patterns across the two models; Firth correction only generated mild shrinkage of effect sizes without changing any core association interpretations reported above.

**Table 5:** Firth-type penalized multinomial logistic regression sensitivity analysis for latent profile membership of dyadic coping among patients with autoimmune diseases and their spouses (*n* = 262).

VVariable	Moderate dyadic coping profile						Spouse-moderate/Patient-low dyadic coping profile						Patient-moderate/Spouse-low dyadic coping profile					
	*β*	Cell *n*	Odds ratio	95% CI		p value	*β*	Cell n	Odds ratio	95% CI		p value	*β*	Cell n	Odds ratio	95% CI		*p* value
				Lower	Upper					Lower	Upper					Lower	Upper	
Patient																		
Age	0.127	—	1.135	0.965	1.338	0.114	0.067	—	1.069	0.919	1.245	0.387	0.232	—	1.261	1.022	1.556	0.031
Illness perception	0.428	—	1.533	1.236	1.903	<0.001	0.687	—	1.989	1.521	2.601	<0.001	0.780	—	2.181	1.533	3.103	<0.001
Marital adjustment	−0.043	—	0.958	0.922	0.996	0.030	−0.067	—	0.936	0.894	0.979	0.004	−0.142	—	0.867	0.800	0.940	<0.001
Economic burden																		
Mild	−1.238	32/24	0.290	0.063	1.338	0.112	−3.603	6/24	0.027	0.004	0.208	<0.001	−3.904	7/24	0.020	0.001	0.314	0.005
Moderate	0.281	64/9	1.325	0.282	6.234	0.722	−1.325	24/9	0.266	0.044	1.608	0.149	−1.915	24/9	0.147	0.012	1.776	0.132
Severe (ref)		20/6	1					29/6	1					17/6	1			
Spouse																		
Age	−0.104	—	0.901	0.788	1.032	0.132	0.164	—	1.178	0.942	1.474	0.163	−0.519	—	0.595	0.454	0.780	<0.001
Marital adjustment	−0.080	—	0.923	0.871	0.978	0.007	−0.174	—	0.841	0.784	0.902	<0.001	−0.299	—	0.741	0.671	0.819	<0.001
Caregiving burden																		
Mild	−1.280	34/22	0.278	0.062	1.255	0.096	−1.961	17/22	0.141	0.022	0.897	0.037	−4.185	7/22	0.015	0.001	0.269	0.004
Moderate	−0.702	42/11	0.495	0.098	2.508	0.396	−0.423	25/11	0.655	0.099	4.346	0.661	−1.903	10/11	0.149	0.012	1.831	0.137
Severe (ref)		40/6	1					17/6	1					31/6	1			
Spouse employment status																		
Employed	−0.005	80/24	0.995	0.226	4.391	0.995	−0.867	42/24	0.420	0.059	2.991	0.387	−0.497	41/24	0.608	0.361	1.024	0.070
Unemployed/retired (ref)		36/15	1					17/15	1					7/15	1			

Reference group = high dyadic coping profile.

Cell *n* denotes the sample size of each categorical subgroup vs. reference group.

Firth-type penalized estimates corresponding to Table 4. All core predictors retained consistent directions and significance with the standard model, supporting robustness.

## 4 Discussion

The present study identified four heterogeneous subtypes of dyadic coping among couples affected by autoimmune diseases, and revealed the differentiated effects of illness perception, marital adjustment, and burden level on subtype membership, thereby providing empirical evidence for the precise stratification of couple-based collaborative interventions.

### 4.1 Overall interpretation of the overall level and profile distribution of dyadic coping in patients with autoimmune diseases and their spouses

In this study, the total dyadic coping scores of both patients with autoimmune diseases and their spouses were at a moderate level, which is generally consistent with findings from studies on couples with brain injury during rehabilitation (29) and older couples with stroke (30). Autoimmune diseases are characterized by unpredictable acute flares, progressive functional impairment, and long-term care needs, sharing substantial similarity with the functional disability and care dependence caused by brain disorders such as brain injury and stroke. According to the systemic transactional model of stress and coping (5), when confronted with long-term, uncontrollable disease-related stress, couples must continuously mobilize cognitive and emotional resources to coordinate their responses; however, the finite nature of these resources makes it difficult to sustain high-intensity collaborative coping over time. Couples therefore tend to adopt conservative, balanced coping strategies, resulting in an overall moderate level of dyadic coping. This also suggests that the coping potential of couples with chronic diseases is generally not fully activated.

Latent profile analysis confirmed that dyadic coping among couples with autoimmune diseases exhibits significant heterogeneity and can be classified into four subtypes, of which 40.1% of couples displayed an asymmetrical and imbalanced coping pattern. This proportion is notably higher than the previously reported 23.7% in couples affected by stroke (31). This differentiated distribution represents a key finding that traditional total-score analysis fails to capture, underscoring the advantage of subtype-based approaches in revealing between-couple differences in coping. According to dyadic coping theory (32), couples under stress constitute an interdependent adaptive unit and should ideally achieve shared coping through collaborative interaction. However, the enduring and relapsing nature of autoimmune diseases, their incurability, and the unpredictability of disease progression impose a unique form of stress that may predispose couples to an imbalance in coping roles, manifesting as mismatched levels of engagement and asymmetric supportive interactions, ultimately resulting in divergent patterns of participation.

### 4.2 Differential effects of multidimensional factors on latent profiles of dyadic coping

Based on latent profile analysis, this study classified the dyadic coping patterns of couples with autoimmune diseases into four categories.

In the High Dyadic Coping Profile, both patients and spouses had the highest scores among the four groups on all positive dimensions, and also scored highest on the negative coping dimension (reverse-scored), indicating that this group of couples was most inclined to cope with disease-related stress in a positive manner, representing an ideal state of dyadic coping.

In the Moderate Dyadic Coping Profile, these couples had stable basic dyadic interaction skills, but showed deficiencies in higher-order coping behaviors such as in-depth stress communication and collaborative problem-solving, leaving considerable room for improvement. This category accounted for the largest proportion and represented the predominant pattern of dyadic coping in couples with autoimmune diseases.

In the Spouse-Moderate/Patient-Low Dyadic Coping Profile, patients scored significantly lower on positive dimensions (e.g., stress communication, supportive coping, and joint coping), while exhibiting more prominent negative coping behaviors. Overall, this pattern reflects a unidirectional imbalance in which patients lack coping motivation and ability, and spouses bear the primary responsibility for care and coping under stress.

In the Patient-Moderate/Spouse-Low Dyadic Coping Profile, spouses scored lower on core positive dimensions such as supportive coping, delegated coping, and joint coping, and showed the most pronounced tendency toward negative coping among the four groups, forming an imbalance characterized by patients’ independent adjustment in the absence of sufficient spousal collaborative involvement.

The following sections will discuss the differential effects of various factors on the different dyadic coping profiles.

#### 4.2.1 Driving effect of illness perception on dyadic coping imbalance

Illness perception was the strongest predictor distinguishing dyadic coping subtypes among couples with autoimmune diseases. Taking the high-level balanced profile as the reference group, every one-point elevation in illness perception was cross-sectionally associated with elevated odds of membership in three unbalanced coping subtypes in ascending order of imbalance severity: the moderate-level balanced profile, the spouse-moderate/patient-low profile, and the patient-moderate/spouse-low profile (OR = 1.683, 2.287, and 3.188, respectively; all *p* < 0.001). This cross-sectional gradient association demonstrates that couples reporting stronger negative illness perceptions tended to exhibit more severe dyadic coping imbalance at a single time point.

We propose a tentative theoretical pathway to interpret this graded correlational pattern (this hypothesized sequential shift requires verification in future longitudinal research): among patients with mild negative illness perceptions, couples generally maintain moderate joint coping capacity. For those with moderately elevated negative illness perceptions, patients may experience reduced coping resources first, corresponding to the spouse-moderate/patient-low subtype observed in this sample. Among patients with pronounced negative illness perceptions, impaired patient coping may further erode spousal coping efficacy via emotional contagion, coping withdrawal and other interpersonal processes, corresponding to the patient-moderate/spouse-low subtype.

This interpretive framework aligns with stress and coping theory, which states that individuals’ cognitive appraisal of stressors acts as a core precursor shaping coping responses and related outcomes. Collectively, these cross-sectional associations further suggest that patients’ subjective illness perceptions, rather than objective disease severity, are strongly linked to disrupted collaborative dyadic coping systems within couples living with chronic autoimmune conditions.

#### 4.2.2 Protective role of marital adjustment in dyadic coping patterns

Both patient and spouse marital adjustment were independent protective factors for the three non-high-level dyadic coping subtypes (all OR < 1). Cross-group comparisons showed that marital adjustment scores progressively decreased from the high-level profile to the patient-moderate/spouse-low profile. The changes in OR values in the regression model further confirmed this gradient association: patient marital adjustment OR decreased from 0.949 (moderate-level profile) to 0.915 (spouse-moderate/patient-low profile) and then to 0.850 (patient-moderate/spouse-low profile); similarly, spouse marital adjustment OR progressively decreased from 0.909 to 0.834 to 0.704. These findings indicate that lower marital adjustment is associated with a progressively higher risk of couples deviating from the high-level dyadic coping pattern, and this protective effect is most pronounced in the unidirectional imbalanced subtypes. As a core emotional bond in couple interaction, marital adjustment can modulate communication and collaborative behaviors under disease-related stress, and thus represents a key protective factor for couples with autoimmune diseases in building a positive dyadic coping pattern.

#### 4.2.3 Effects of patient and spouse age on dyadic coping profiles

Patient age was a significant positive predictor for the Patient-Moderate/Spouse-Low Dyadic Coping Profile (OR = 1.323, 95% CI: 1.020–1.717, *p* < 0.05), indicating that older patient age was independently associated with a higher probability of belonging to this imbalanced pattern after adjusting for other covariates. This association may be explained by age-related accumulation of family and occupational role burdens; when combined with the long-term stress of a chronic disease, these multiple demands may exacerbate patients’ feelings of loss of control over the illness and negative illness perceptions (33). At the same time, the spouses in this group had the lowest mean age among the four groups, which may result in insufficient experience in disease caregiving and limited dyadic collaboration skills, and thus inability to provide adequate family support (34). Future longitudinal studies could verify this mechanistic pathway.

Spouse age was a significant negative predictor for the Patient-Moderate/Spouse-Low Dyadic Coping Profile (OR = 0.662, 95% CI: 0.452–0.969, *p* < 0.05), indicating that older spouse age was associated with a lower probability of the couple belonging to this imbalanced pattern. Compared with younger spouses, older spouses generally possess more mature emotion regulation skills and more experience in handling family stress. When facing patients’ high negative illness perceptions and emotional distress, older spouses are better able to maintain stability in their own coping state, thereby avoiding impairment of their own coping function and mitigating the imbalance in dyadic interaction.

#### 4.2.4 Threshold effects of economic burden and caregiving burden on dyadic coping

Economic burden and spousal caregiving burden are key practical factors influencing dyadic coping in affected couples. Using severe burden as the reference category, the results showed that mild economic burden and mild caregiving burden were significant negative predictors across all non-high-level dyadic coping subtypes, effectively reducing the risk of couples falling into coping imbalance. In the Spouse-Moderate/Patient-Low Profile, mild patient economic burden (OR = 0.015) and mild spouse caregiving burden (OR = 0.113) showed significant protective effects; in the Patient-Moderate/Spouse-Low Profile, the corresponding OR values were 0.009 and 0.004, respectively, also demonstrating stable protective effects. In contrast, moderate burden had no significant predictive value for any of the imbalanced subtypes. These findings suggest that the effect of burden on dyadic coping may follow a threshold effect rather than the traditionally assumed single linear negative correlation (33). Only when the family’s disease-related burden subsides to a mild level can couples maintain a favorable high-level dyadic coping state. Once burden accumulates to a moderate or higher level, dyadic coping function may suffer persistent impairment, and slight improvements in burden are unlikely to reverse the established imbalance pattern.

### 4.3 Nursing implications

Couples in the High Dyadic Coping Profile represent the optimal family stress adaptation subtype among couples with autoimmune diseases. The nursing goal is to consolidate their strengths and leverage their exemplary effect. It is recommended that these couples serve as positive family coping models, and their experiences in bidirectional communication and mutual empowerment be systematically identified. Given the high unpredictability of autoimmune disease recurrence, prospective emergency education and long-term chronic disease management guidance should be provided, supplemented by advanced dyadic coping training such as in-depth stress communication and shared disease decision-making, to continuously consolidate the couple‘s positive family interaction system. The feasibility of such couple-oriented approaches is supported by existing evidence in related conditions. Carberry et al. (35) conducted a qualitative systematic review and metasynthesis of couples’ experiences of coping with multiple sclerosis (MS), identifying four core themes: “dance of accommodation,” “a sense of unity,” “outside of us,” and “evolving as a unit,” which reveal the intrinsic mechanism by which couples reframe the disease as a “shared challenge” to facilitate collaborative coping in the long-term management of MS. Additionally, a systematic review of dyadic psychosocial interventions for couples affected by MS confirmed potential positive effects on partnership quality and quality of life (36). These findings suggest that couple-based intervention approaches, which have shown feasibility in MS, may also be applicable to autoimmune disease populations.

The Moderate Dyadic Coping Profile accounted for the largest proportion. Clinically, cognitive-behavioral interventions can be used to address and modify irrational illness-related cognitions, combined with couple-based cognitive restructuring training to narrow cognitive discrepancies about the disease between partners and prevent the progressive erosion of dyadic coping by negative cognitions. In addition, moderate-to-severe burden should be used as a high-risk screening cut-off. Family economic pressure can be alleviated by optimizing chronic disease management during stable phases and standardizing follow-up discharge procedures. A multidimensional social support system should be constructed based on supportive care theory, employing strategies such as respite care and group psychological interventions to alleviate caregiver burnout. Furthermore, drawing on the TCU program developed by Lyons et al. (37), seven video-based sessions can be designed, covering team awareness building, disease and role clarification, collaborative management communication, partner health support, relationship strengthening, and long-term plan development, to enhance the dyadic coping level of couples.

For couples in the Spouse-Moderate/Patient-Low Dyadic Coping Profile who exhibit a rigid, full-care inertia pattern, the couple-based collaborative intervention program developed by Yang (38) based on King’s goal attainment theory can be adapted. Through establishing shared goals, collaborative care planning, and joint decision-making exercises, spouses can be guided to implement empowering care and mobilize patients’ motivation for autonomous disease management. Individualized cognitive-behavioral interventions (e.g., cognitive restructuring, behavioral activation) should be implemented for patients to ameliorate negative cognitions related to loss of disease control and hopelessness about prognosis, thereby reducing passive coping. Simultaneously, couple-based emotional enhancement should be strengthened to compensate for the erosion of family interaction caused by long-term unidirectional care, fostering a bidirectional collaborative coping model.

The Patient-Moderate/Spouse-Low Dyadic Coping Profile is most prevalent among young and middle-aged couples. Research by Hou (10) has demonstrated that active coping by one partner has a significant positive cross-partner effect on the other partner’s relationship satisfaction, highlighting the crucial role of activating bidirectional dyadic coping in improving adaptation outcomes for both partners. Based on this, role adjustment and cognitive restructuring interventions should be provided for young and middle-aged patients to alleviate negative cognitions induced by multiple role stress. For young spouses, a dedicated empowerment system should be established, covering chronic disease management knowledge, supportive communication, and collaborative coping training. Structured emotional interventions should also be implemented to activate the protective role of marital adjustment.

From a public health and gerontological perspective, the four latent dyadic coping profiles carry actionable implications consistent with the journal’s scope. First, the asymmetric subtypes (Spouse-Moderate/Patient-Low and Patient-Moderate/Spouse-Low) account for a substantial proportion of couples, imposing hidden psychological and caregiving burdens on middle-aged and older adults with autoimmune diseases and their spouses. Persistent unilateral caregiving accumulates chronic distress, heightens marital strain, impairs quality of life, and increases healthcare utilization. Second, this four-profile typology can inform standardized screening in primary care. General practitioners and community nurses could administer brief dyadic coping assessments during routine chronic disease follow-ups to detect at-risk couples before caregiver burnout or marital dysfunction occurs. Third, these findings support policies for informal caregivers. Health administrations could integrate couple-focused coping training into community chronic disease management, expand respite care, and subsidize psychological support programs to alleviate households’ financial and psychological strain. Lastly, tailored health education for middle-aged and older couples with autoimmune diseases should promote mutual collaborative coping and reduce unilateral care patterns, thereby strengthening family-based resilience in later life.

## 5 Limitations

The present study has several limitations. First, the cross-sectional design precludes causal inference and cannot verify any temporal or sequential progression among different dyadic coping subtypes; all correlations reported herein merely reflect associations captured at a single time point. Accordingly, any progressive pattern hypothesized in the Discussion should be interpreted as a tentative theoretical proposal requiring future longitudinal verification. Additionally, the questionnaire did not capture other chronic stressors (e.g., physical pain, sleep disturbance, occupational stress, and social stigma) that may deplete couples’ coping resources. Second, convenience sampling from three tertiary hospitals may limit generalizability to community and primary care settings. Third, sample sizes across disease subtypes were uneven (e.g., only 27 cases of systemic sclerosis), and the high-level balanced profile identified by LPA was relatively small (39 cases, 14.9%), which may widen confidence intervals and reduce precision in regression analyses. Fourth, medication use (type, duration, prescription status) was not recorded, nor were potential side effects assessed, leaving this confounder unadjusted. Fifth, excluding those with prior psychological intervention may remove both highly distressed and well-adjusted extreme subgroups, introducing selection bias that could either overestimate or underestimate true adaptation levels, thus limiting generalizability to clinically referred populations.

## 6 Conclusion

Based on the theoretical framework of dyadic coping, this study used latent profile analysis to identify four heterogeneous subtypes of dyadic coping in patients with autoimmune diseases and their spouses, along with their core influencing factors. This classification approach overcomes the limitations of traditional total score-based assessment models. Nursing interventions for families with autoimmune diseases should abandon a one-size-fits-all model and instead implement stratified, targeted couple-based interventions according to the specific coping subtype of each couple, starting from multiple pathways including illness cognitive restructuring, emotional relationship maintenance, and burden level management, so as to more effectively enhance family dyadic coping capacity and long-term disease adaptation outcomes.

## 7 Future directions

Given the cross-sectional design of this study, causal inferences cannot be drawn. Future longitudinal follow-up studies are needed to further investigate the dynamic effects of factors such as illness perception, marital adjustment, and family burden on the transitions between dyadic coping subtypes, and to identify the evolutionary pathways and critical turning points among the different subtypes. Second, based on the four identified subtype characteristics and their influencing factors, future research could develop and validate stepped nursing interventions targeting couples, and systematically evaluate their effectiveness through randomized controlled trials. Additionally, in clinical nursing practice, early identification of high-risk imbalanced couples is recommended to advance the timing of precise nursing interventions, thereby improving family disease adaptation capacity and long-term health outcomes.

## Data Availability

The raw data supporting the conclusions of this article will be made available by the authors, without undue reservation.
